# Sicca syndrome as complication of COVID‐19 infection: A case report

**DOI:** 10.1002/ccr3.9177

**Published:** 2024-07-16

**Authors:** Raghad Tarcha, Naram Khalayli, Maysoun Kudsi

**Affiliations:** ^1^ Department of Rheumatology, Faculty of Medicine Damascus University Damascus Syria; ^2^ Department of Psychiatry, Faculty of Medicine Damascus University Damascus Syria; ^3^ Faculty of Medicine Damascus University Damascus Syria

**Keywords:** COVID‐19 infection, dry eye, dry mouth, sequela, sicca syndrome

## Abstract

Several reports of suspected oral and ocular manifestations of coronavirus disease 2019 (COVID‐19) has prompted investigations into ocular signs, symptoms, and transmission (5).11.2% of patients with COV19 infection had ocular symptoms, including ocular pain, conjunctivitis, dry eye, and floaters, meanwhile, many studies had documented oral symptoms such as dry mouth and dysgeusia in these patients. Our case reported a 39‐year‐old male, presented with symptoms of dry mouth and dry eye lasting more than 3 months. The patient had recovered from (PCR‐confirmed) COVID‐19 which lasted 10 days, 4 months ago. The physical examination was normal. Ocular findings include conjunctival hyperemia and superficial punctate keratitis. The anti‐nuclear antibody (ANA) was weekly positive at 1/80. Schirmer test considered positive. He continued on 200 mg/day of hydroxychloroquine, along with tear drops until now with remission. Sicca symptoms may be a sequel of COVID‐19 infection, and physicians should be aware of this sequel. The sequela of this infection is not understood, with limited data in the literature. Future prospective cohort studies are needed to reveal the impact of these features on oral health.

## 1 INTRODUCTION

In the last two decades, this is the third deadliest coronavirus pandemic, severe acute respiratory syndrome coronavirus 2 (SARS‐CoV‐2), resulting in increased morbidity and mortality associated with the acute phase.^1^ Several symptoms were reported in the post‐acute COVID‐19 syndrome including dyspnea, chest pain and arthralgia, and cognitive disturbances.^2^

The post‐acute COVID‐19 syndrome can be classified as acute, subacute, and chronic symptomatic COVID‐19. Chronic COVID‐19 syndrome is the observed symptom after 12 weeks of acute onset and is not due to another diagnosis. Fatigue and sleep difficulties are the most frequently reported symptoms.^3^

Mechanisms that contributed to the pathophysiology of post‐acute COVID‐19 were reported by studies. It was suggested a multifactorial mechanism including viral pathophysiologic changes, immunologic aberrations, acute phase response inflammation, and post‐intensive care syndrome sequela. SARS‐CoV‐2 infection in patients with comorbidities may have excessive release of cytokines and cytokine storm, resulting in immune destruction.^4^ Several reports of suspected ocular manifestations of coronavirus disease 2019 (COVID‐19) have prompted investigations into ocular signs, symptoms, and transmission.^5^

## 2 CASE HISTORY/EXAMINATION

A 39‐year‐old Syrian male, a nonsmoker, presented in June 2023, to the Rheumatology Department in Al‐Muwassat University Hospital, Damascus, Syria, complaining of symptoms of dry mouth and dry eye lasting more than 3 months. The patient had recovered from (PCR‐confirmed) COVID‐19 which lasted 10 days, 4 months ago. He had no previous medical, surgical history, comorbidities, or positive family history.

The physical examination was normal. Ocular findings include conjunctival hyperemia and superficial punctate keratitis.

## 3 METHODS

The immune profile included: rheumatoid factor, anti‐cyclic citrullinated peptide antibody, Anti‐La, Anti Ro, perinuclear antineutrophil cytoplasmic antibody, antineutrophil cytoplasmic antibody were negative. The anti‐nuclear antibody (ANA) was weekly positive at 1/80. Complements were within normal limits. Schirmer test considered positive for severe dry eye (≤5 mm/5 min). The resting salivary flow was 0.07 mL/min. The salivary gland biopsy was normal.

## 4 CONCLUSION AND RESULTS

He was treated with artificial tears, and cholinergic agonists for 3 months without remission of his dryness symptoms, so 20 mg/day of prednisolone was added, in addition to 200 mg/day of hydroxychloroquine. Six weeks later, on a routine follow‐up, his symptoms improved significantly, so we began to taper the prednisolone dose. He continued on 200 mg/day hydroxychloroquine, along with tear drops until now.

## 5 DISCUSSION

Several reports of suspected ocular manifestations of coronavirus disease 2019 (COVID‐19) has prompted investigations into ocular signs, symptoms, and transmission.^5^ A total of 11.2% patients with COVID‐19 infection had ocular symptoms, including ocular pain, conjunctivitis, dry eye, and floaters according to a meta‐analysis involving 1533 patients.^5^ Xerostomia was reported as a symptom in COVID‐19 individuals.^6^

Patients infected with SARS‐CoV‐2 can present with eye redness, ocular irritation, eye soreness, foreign body sensation, tearing, mucoid discharge, eyelid swelling, congestion, and chemosis. These symptoms have more commonly affected patients with severe systemic symptoms of COVID‐19, though they can rarely present as an initial manifestation of the disease.^7^ Examination findings include unilateral or bilateral bulbar conjunctiva injection, follicular reaction of the palpebral conjunctiva, watery discharge, and mild eyelid edema.^7^ A case report published by Cheema et al. described the first case of keratoconjunctivitis as the presenting manifestation of COVID‐19 in North America.^8^ Many studies had documented oral symptoms such as dry mouth and dysgeusia in patients with COVID‐19 infection, caused by SARS‐CoV‐2 binding to ACE‐2 receptors on the epithelium of salivary glands, replicate, lyse cells, and propagate signs and symptoms, resulting in chronic sialoadenitis, or from the neurotoxic effects of this virus on target organs, and CNS.^9^ The cause for this association is unclear and may include autoimmunity causes, the increased risk for infection and the impact of other comorbidities and therapies.^7^, ^8^, ^9^, ^10^

Interestingly, Costa et al. did not consider oral dryness an aspect of post‐COVID‐19 syndrome,^11^ which describe the medium‐term ophthalmological findings in patients recovering from COVID‐19. Patients recovered from the acute phase of COVID‐19 underwent a complete ophthalmological evaluation. They found the median presenting visual acuity was 0.1 (0–0.2) and BCVA 0 (0–0.1).^12^ Anterior segment biomicroscopy was unremarkable, except for dry eye disease, verified in 10.9% of them. Xerostomia has been reported as a relevant symptom of COVID‐19 individuals,^13^ but xerophthalmia has not been associated with this disease.^11^ Nevertheless, an impressive elevation of Sjögren's syndrome notification^14^ during the COVID‐19 pandemic in Brazil. Interestingly, oral dryness was not considered a relevant aspect of post‐COVID‐19 syndrome by Costa et al.,^11^ even though their population was less than 30% of critical COVID‐19 cases.^11^ Notwithstanding, whether this sicca syndrome represents actual Sjögren's syndrome should be better evaluated. Interestingly, a post‐COVID‐19 syndrome in patients with primary Sjögren's syndrome has a frequency of nearly 60% and is associated with hospitalization, baseline CRP levels, and hydroxychloroquine levels, but worsened sicca symptoms were not reported.^12^ Also, the impact of these features on oral health should be a matter of concern for future prospective cohort studies. Vlachoyiannopoulos et al. reported that 69% of patients were found to have one of the following autoantibodies post‐COVID‐19 infection: ANA, anti‐cardiolipin, anti‐beta‐2‐glycoprotein‐1, antineutrophil cytoplasmic antibodies, and anti‐cyclic citrullinated peptide. The most frequently observed antibodies in these patients were ANA, aCL, and anti‐β2GP1.^14^

Sicca symptoms may be a sequel of COVID‐19 infection, and physicians should be aware of this sequel. The sequela of this infection is not understood, with limited data in the literature. Future prospective cohort studies are needed to reveal the impact of these features on oral health.

## AUTHOR CONTRIBUTIONS

**Raghad Tarcha:** Conceptualization; data curation; writing – original draft. **Naram Khalayli:** Conceptualization; data curation; writing – original draft. **Maysoun Kudsi:** Conceptualization; data curation; writing – original draft; writing – review and editing.

## FUNDING INFORMATION

No funding.

## CONFLICT OF INTEREST STATEMENT

The authors declare no conflicts of interest.

## ETHICS STATEMENT

This case report was according to the Declaration of Helsinki. The ethical approval for this report was not necessary.

## CONSENT

Written informed consent was obtained from the patient to publish this report in accordance with the journal's patient consent policy.

## Data Availability

Not applicable.
